# Single-step genomic BLUP (ssGBLUP) effectively models small cattle populations: lessons from the Israeli-Holstein Herdbook

**DOI:** 10.1186/s12864-024-11074-8

**Published:** 2024-11-27

**Authors:** Arie Yehuda Curzon, Ephraim Ezra, Joel Ira Weller, Eyal Seroussi, Vinzent Börner, Moran Gershoni

**Affiliations:** 1 ARO, The Volcani Center, Rishon LeZion, 15159 Israel; 2 Robert H. Smith Faculty of Agriculture, Food and Environment, Hebrew University of Jerusalem, Rehovot, 76100 Israel; 3 Israel Cattle Breeders Association, Caesarea, 38900 Israel; 4 GHPC Consulting and Services PTY LTD, Armidale, N.S.W Australia

**Keywords:** APEX- Linear mixed Model Software, Dairy cattle, Genomic evaluation, Israeli holsteins, Inflation

## Abstract

**Background:**

Routine genomic-estimated breeding values (gEBVs) are computed for the Israeli dairy cattle population by a two-step methodology in combination with the much larger Dutch population. Only sire genotypes are included. This work evaluated the contribution of cow genotypes obtained from the Israeli Holstein population to enhance gEBVs predictions via single-step genomic best-linear unbiased prediction (ssGBLUP). The gEBV values of 141 bulls with daughter information and high reliabilities for 305-day lactation yield of milk, fat, and protein were compared with the bulls’ predicted ssGBLUP-gEBVs using a truncated dataset omitting production data of the last five years. We investigated how these sire gEBVs were affected by varying polygenic weights in the genomic relationship matrices and by deleting old phenotypic or genotypic records.

**Results:**

The correlations of the predicted gEBVs for milk, fat and protein computed from the truncated data with the current gEBVs based also on daughter records of the last five years were 0.64, 0.57, and 0.56, respectively, for a polygenic weight of 0.5, similar to the values achieved by the current two-step methodology. The regressions of the current gEBVs on the predicted values were 0.9 for milk and 0.7 for fat and protein. Genotyping of 1.8-5 cows had the approximate statistical power of one additional bull depending on the trait. Omitting phenotype records earlier than 2000 resulted in similar gEBV values. Omitting genotypes before 1995 improved the regression coefficients. For all experiments, varying the polygenic weights over the range of 0.1 to 0.9 resulted in a trade-off between correlations and overestimation of gEBVs for young bulls.

**Conclusions:**

The model suffers from overestimation of the predicted values for young bulls. The time interval used for inclusion of genotypic and phenotypic records and adjustment of the polygenic weight can improve gEBV predictions and should be tuned to fit the tested population. For relatively small populations, genotyping of cows can significantly increase the reliability of gEBVs computed by single-step methodology. By extrapolation of our results, records of ~ 13,000 genotyped cows should provide a sufficiently large training population to obtain reliable estimates of gEBVs using ssGBLUP.

**Supplementary Information:**

The online version contains supplementary material available at 10.1186/s12864-024-11074-8.

## Introduction

Estimated breeding values (EBVs) for dairy cows have been used for breeding since their development by Henderson in the 1950s. Since the 1970s, the Best Linear Unbiased Prediction (BLUP) method has been used to estimate EBVs based on a pedigree relationship matrix (A-matrix, ABLUP) [1]. With the advent of high-throughput genotyping based on thousands of genetic markers, VanRaden [2] proposed a method to utilize genomic data for genetic prediction by computing genetic relationship matrices, which were based on DNA markers. In the first years of genomic evaluations, the costs per individual genotyped were relatively high, and only progeny-tested sires were genotyped. VanRaden’s algorithm uses a two-step procedure based on the computation of a Genomic Relationship Matrix (G-matrix) among genotyped bulls. The EBVs are first calculated using the A-matrix for the entire population. In the second step, deregressed evaluations (DD) or daughter yield deviations (DYD) of sires are used to calculate direct genomic values (DGV) for bulls with genomic information. Combining the DGV with the parent average or with pedigree-based EBVs yields genomic EBV (gEBV) [2]. Shortly thereafter, a single-step algorithm, ssGBLUP, was proposed [3, 4]. The single-step method combines pedigree, genomic, and phenotypic information from all individuals to simultaneously calculate gEBVs, even though only a small fraction of the population is genotyped. Whereas the two-step method uses the G-matrix as a kinship matrix, the single-step method utilizes the H-matrix [3]. The ssSNPBLUP method is an equivalent variant of ssGBLUP. With ssSNPBLUP, millions of genotyped animals can be analyzed without approximating genomic relationships [5, 6].In recent years, with the development of high-throughput genotyping, genomic selection has become routine for dairy cattle [7].

The number of genotyped animals and the heritability of traits are key factors for accurate predictions of gEBVs [8, 9]. Thus, some relatively small dairy populations combine their data with other, larger populations to increase the number of genotyped bulls [10–12]. However, populations from different geographical regions can significantly differ in size, recording methodologies, trait definitions, environmental conditions, and selection objectives, which should decrease the accuracy of prediction [11]. As genotyping costs have decreased, millions of cows with production records have been genotyped (https://uscdcb.com/database-stats/). The inclusion of genomic data from cows should improve the accuracy of evaluations [13, 14]. However, since cows’ gEBVs are chiefly based on their own performance, the reliability of their gEBVs is typically lower than that of bull gEBVs based on many daughter records. Moreover, the effect of the inclusion of cow genotypes in genomic evaluations by the two-step methodology is not straightforward, because of double sampling and the overestimation of cow performance due to preferential treatment of genotyped cows [15–17]. Although studies have suggested ways to overcome these challenges for two-step algorithms [16, 18], combining cow data in the ssGBLUP algorithm is straightforward [15, 19]. Genotyped cows in the multi-step method may even decrease the accuracy of gEBVs and increase bias [19]. The incorporation of cow genotypes in the single-step method can increase prediction accuracy and reduce bias [19]. The gain of using cow genotypes could be highly beneficial in small populations but may be negligible in large populations [19]. The gain obtained is a function of the heritability of the trait, the sample size and pedigree relationship [14, 19].

In the Israeli Holstein population ~ 30,000 milk recorded cows are produced each year, but only 40–50 bulls are progeny tested. Since 2013, the Israel Cattle Breeders’ Association (ICBA) in collaboration with CRV of the Netherlands computes gEBVs for routine evaluation based on a two-step algorithm [11]. Lourenco et al. [20] estimated ssGBLUP evaluations for the Israeli dairy population including cow genotypes. In that analysis, the total number of genotypes was insufficient to obtain evaluations with useful accuracies. In the last decade, the number of genotyped Israeli cows has substantially increased, providing an opportunity to improve gEBVs. Therefore, in this study, we evaluated the contribution of cow genotypes to gEBV estimation by the ssSNPBLUP method in the Israeli Holstein dairy cattle population for three yield traits: milk kg, fat kg, and protein kg over 305-day lactations. We investigated changes in sire gEBVs and their reliability from truncated datasets under different polygenic weights. Evaluations of young sires derived by ABLUP, the two-step procedure and ssSNPBLUP were compared. Genomic evaluations are based on population-wide linkage relationships between genetic markers and causative polymorphisms. Allelic frequencies and linkage relationships change over time and should affect the usefulness of “old” genotypes [12, 21]. We therefore tested the effects of excluding old phenotypes recorded before 1990 or old genotypes obtained before 1995 and 2000.

## Results

### Descriptive statistics

The descriptive statistics of the datasets analyzed are given in Table 1. For all three production traits the genetic and residual variance components based on a dataset containing all individuals born from 2010 to 2014 are given in Table S1. The heritability estimates and their standard errors are given in Table 2. These values were slightly higher than earlier heritabilities reported for this population [22]. This is likely due to more accurate trait recording and pedigree data. Heritabilities decreased with increase in parity for all three traits.

**Table 1:** Description of the datasets

Dataset	DS90-19	DS90-23	DS00-19	DS00-23	DS10-23
Calving years^1^	1990–2019	1990–2023	2000–2019	2000–2023	2010–2023
Birth year (mean)	2000	2003	2004	2007	2011
Analysis purpose^2^	Prediction	Observation	Prediction	Observation	REML
Cows in pedigree	1,042,049	1,189,601	762,223	913,507	611,801
Bulls in pedigree	3,529	3,809	2,627	3,202	3,083
Cows with records	897,792	1,043,405	613,233	758,846	390,896
Genotyped animals (cows/bulls)					
All	8,259/1,812	8,260/1,876	8,259/ 1,731	8,260/1,795	NR^3^
With records^4^	5,207/1,552	8,260/1,876	5,207/1,471	8,260/1,795	NR
From 1995^5^	NR	NR	8,259/1,297	8,260/1,361	NR
From 2000^6^	NR	NR	8,259/1,100	8,260/1,164	NR

^1^ The calving years range of the documented cows in the dataset

^2^ Prediction = production records generated after 2019 were deleted, Observation = records generated after 2019 were included

^3^ NR = not relevant

^4^ The number of genotyped animals with phenotypic records (cows) or phenotypic daughter records (bulls)

^5^ These datasets were denoted DS00-19(95) and DS00-23(95) when genotypes before 1995 were excluded

^6^ These datasets were denoted DS00-19(00) and DS00-23(00) when genotypes before 2000 were excluded

**Table 2:** Heritability and standard error (SE) values for all variables calculated from the estimated REML variance component analysis

Lactation number	Milk	SE	Fat	SE	Protein	SE
1	0.47	0.003	0.45	0.003	0.41	0.003
2	0.36	0.003	0.38	0.003	0.32	0.003
3	0.29	0.004	0.32	0.004	0.26	0.004
4	0.25	0.005	0.27	0.005	0.22	0.005
5	0.21	0.007	0.23	0.007	0.19	0.007

**Fig. 1:**
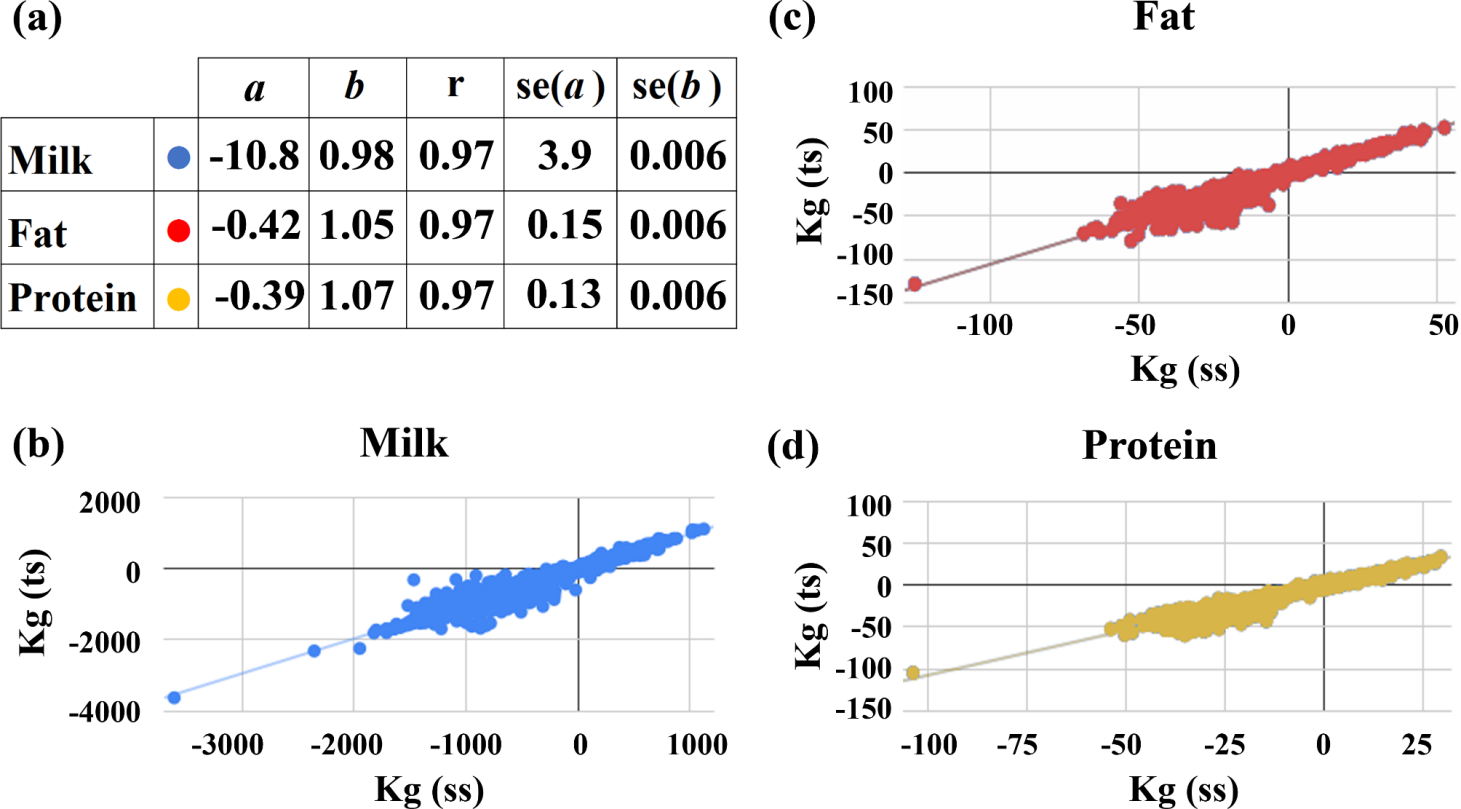
Linear regression analysis between PTA values of the two-step and ssSNPBLUP methods. Regression analysis of the PTA of 1,858 bulls with a reliability ≥ 0.75, computed by the two-step method (ts) and the ssSNPBLUP method (ss), for milk, fat and protein yield from the DS00-23 dataset. The regression statistics are given in panel (**a**): *a* = y-intercept; *b* = regression coefficient; r = correlation; se = standard error. The regressions and the individual bull values are given in the (**b**), (**c**) and (**d**) panels

We first compared the predicted transmission ability values (PTAs, PTA = gEBV/2) from ssSNPBLUP computed from the DS00-23 dataset (Table 1) to the corresponding PTAs scores obtained by the current two-step GBLUP for 1858 bulls with reliability values > 0.75 in the February, 2024, evaluation. Results are given in Fig. 1. Correlations of 0.97 were observed between PTAs calculated from ssSNPBLUP and PTAs calculated from the current two-step GBLUP for all three production traits. The means for the PTA values obtained from ssSNPBLUP were slightly greater, as can be deduced from the negative y-intercept values. Although the slopes ranged between 0.98 and 1.07, all three slopes were significantly different from unity, and all the y-intercepts were significantly different from zero.

### Performance of the different methods

**Fig. 2:**
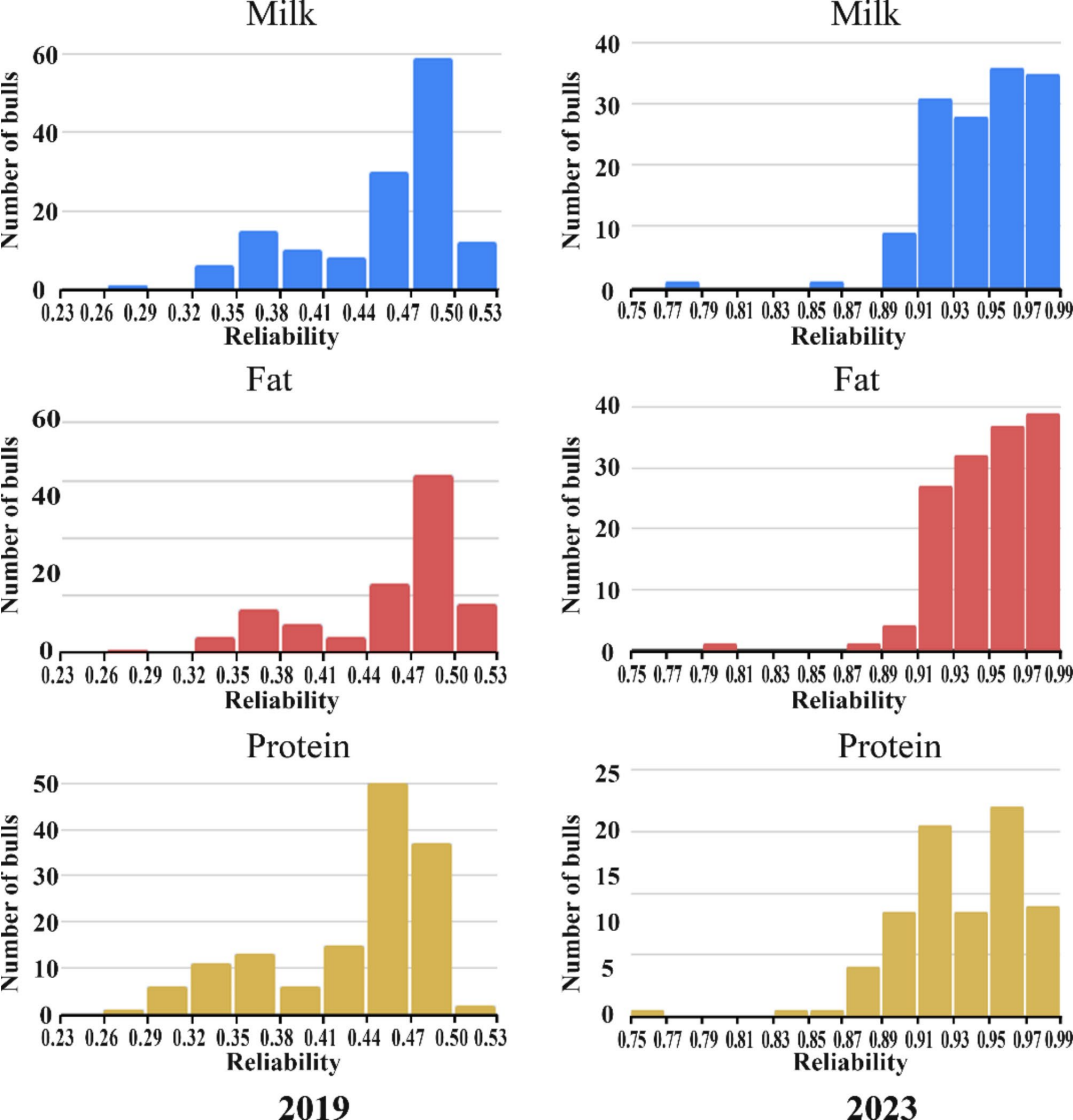
Distribution of reliabilities for a set of 141 bulls. The reliabilities of ssSNPBLUP were calculated from the truncated data (DS00-19, left panels) and the complete data (DS00-23, right panels) with a polygenic weight of 0.5

To assess the effect of different models integrating cow genotypes on the prediction accuracy of the ssSNPBLUP method, we selected a set of 141 sires whose first daughters calved between 2019 and 2023, with current reliabilities *≥* 0.75 for all three traits. Of these bulls, 139 had evaluations in 2019 by the two-step methodology. True reliabilities were obtained by inverting the single-step mixed-model coefficient matrix (Fig. 2) for the gEBVs computed by ssSNPBLUP with DS00-23 (Table 1). For these 141 bulls, in each dataset (Table 3), the PTAs were calculated from the complete datasets (DS90-23 and DS00-23; Table 1) and regressed against the PTAs of these bulls, as computed from the truncated datasets (DS90-19 and DS00-19, respectively, Table 1), which included only daughter records up to 2019. As very few bulls were genotyped before 2000, this design allowed us to test the effect of animals with records but without genotypes on the prediction accuracy of the evaluations.

Except for the pedigree-based BLUP (Experiment 1, ABLUP, Table 3), genotype information was included in all other analyses, which improved predictions. Both GBLUP methods provided better predictions than ABLUP for all three traits with respect to both correlations and regressions. Correlations for the ssSNPBLUP method were equal or greater than those for the two-step method, which combines bull genotypes from the Israeli and Dutch populations, for fat and protein but not for milk. Experiments 2 and 3 showed the highest correlations for milk and protein but not fat (Table 3).

**Fig. 3:**
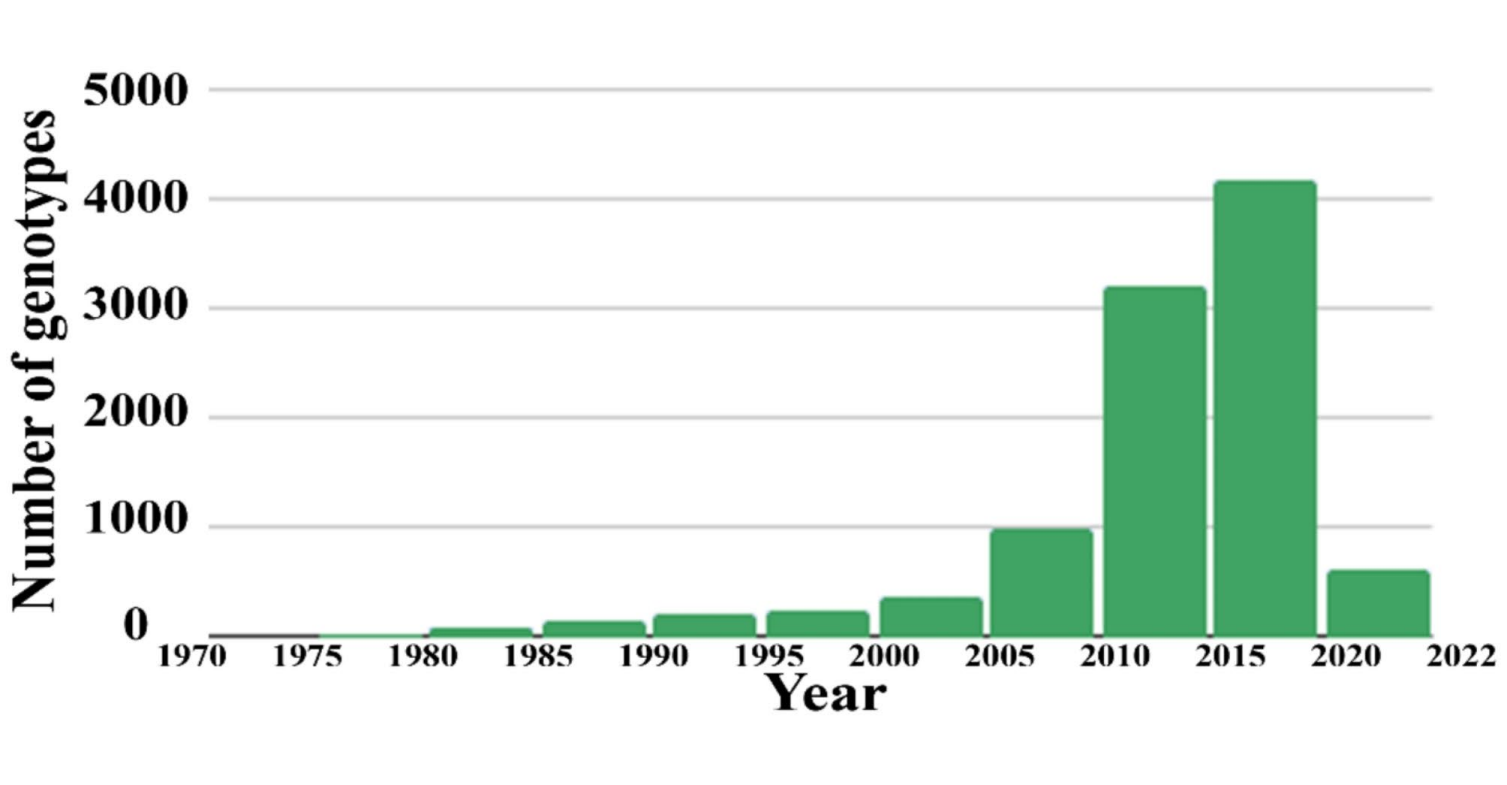
Distribution of the number of genotyped animals per birth year. A bar chart representing a frequency distribution was plotted based on the genotyped animals in the DS90-23 dataset (Table 1)

The inclusion of records starting in 1990 (DS90-23 and DS90-19, Experiment 2, Table 3) did not improve the prediction. Only 81 additional genotyped animals were included compared to the other datasets with records starting in 2000 (DS00-23 and DS00-19, Experiment 3, Table 3). As shown in Fig. 3, genotyping was minimal before 2000. Most of these animals were bulls returned to general service based on their progeny test results and are therefore not representative of the general population.

For all ssSNPBLUP experiments, we observed overestimation of the PTA values for the 141 tested bulls, as indicated by regression coefficients less than unity (Table 3; Tables S2-S3). Specifically, this trend was most extreme for the regression coefficients of fat and protein, which were significantly different from unity for all experiments (*p* < 0.05, Table 3). Regression coefficients were greater for datasets that excluded genotypes from individuals born before 1995 (DS00-19(95), Experiment 4, Table 3) or 2000 (DS00-19(00), Experiment 5, Table 3) under all tested polygenic weights (Table 3 and Tables S2-S3), and the correlations were similar. Thus, considering both regression coefficients, omitting the genotypes of individuals born before 1995 was advantageous (Experiment 4, Table 3, Table S3). The regressions for ABLUP (Experiment 1, Table 3) were lower than the ssSNPBLUP regressions for all the experiments, but the regressions obtained by the two-step method (Experiment 6, Table 3) were closer to unity for all 3 traits. The variance of predictors was greater in ssSNPBLUP than in the two-step method, which may explain the difference in regression coefficients. For the 139 predictor bulls included in both methods, the variances were 56,486, 74.52, and 23.94 and 82,416, 100.02, and 46.1 for milk, fat and protein, respectively, in the two-step (Experiment 6, Table 3) and ssSNPBLUP methods (Experiment 3, Table 3). Thus, variances of the predictor bulls were higher by ssSNPBLUP for all three traits, and nearly double for protein. The correlation values of the predictions between the two methods were 0.71, 0.64 and 0.57 for milk, fat and protein, respectively. Interestingly, averaging the two-step and ssSNPBLUP methods (Experiment 7, Table 3) gave the best prediction results with respect to both regressions and correlations for all three traits.

**Table 3:** Regression parameters for all experiments

Trait	Experiment	Analysis	Datasets^1^	a^2^	b	*r*	se(b)	se(a)
Milk kg	1	ABLUP	DS00-19 vs. DS00-23	-28.78	0.82	0.4	0.16	80.44
	2	ssSNPBLUP	DS90-19 vs. DS90-23	-141.28*	0.83*	0.64	0.08	57.68
	3	ssSNPBLUP	DS00-19 vs. DS00-23	-140.48*	0.84*	0.64	0.08	58.02
	4	ssSNPBLUP	DS00-19(95) vs. DS00-23(95)	-129.97*	0.87	0.63	0.09	57.31
	5	ssSNPBLUP	DS00-19(00) vs. DS00-23(00)	-117.69*	0.89	0.62	0.1	57.79
	6	Two-step method	DS90-19 vs. DS90-23	-169.6**	1.1	0.7	0.1	52.5
	7	Averaged^3^	DS00-19 vs. DS00-23	-203.62***	1.1	0.71	0.09	55.41
Fat kg	1	ABLUP	DS00-19 vs. DS00-23	7.41*	0.56***	0.36	0.12	3
	2	ssSNPBLUP	DS90-19 vs. DS90-23	1.78	0.67***	0.56	0.08	1.94
	3	ssSNPBLUP	DS00-19 vs. DS00-23	1.28	0.67***	0.57	0.08	2.59
	4	ssSNPBLUP	DS00-19(95) vs. DS00-23(95)	1.13	0.7***	0.58	0.08	2.5
	5	ssSNPBLUP	DS00-19(00) vs. DS00-23(00)	1.91	0.7**	0.56	0.09	2.51
	6	Two-step method	DS90-19 vs. DS90-23	1.21	0.8*	0.56	0.1	2.8
	7	Averaged	DS00-19 vs. DS00-23	-2.73	0.89	0.63	0.09	2.66
Protein kg	1	ABLUP	DS00-19 vs. DS00-23	7.87**	0.47**	0.21	0.19	2.73
	2	ssSNPBLUP	DS90-19 vs. DS90-23	1.78	0.67***	0.56	0.08	1.94
	3	ssSNPBLUP	DS00-19 vs. DS00-23	1.37	0.68**	0.56	0.09	2
	4	ssSNPBLUP	DS00-19(95) vs. DS00-23(95)	1.46	0.71**	0.54	0.09	2.01
	5	ssSNPBLUP	DS00-19(00) vs. DS00-23(00)	1.77	0.73**	0.52	0.1	2.05
	6	Two-step method	DS90-19 vs. DS90-23	0.2	0.9	0.51	0.13	2.4
	7	Averaged	DS00-19 vs. DS00-23	-2.23	0.98	0.59	0.11	2.27

^1^ Datasets are described in Table 1. Parameters were obtained by comparing the Predicted Transmitting Abilities (PTAs) computed from the truncated (predicted) and complete (observed) datasets for a set of 141 bulls. Results are presented for polygenic weight of 0.5. For full experiments results see supplementary tables. For the current two-step method (Experiment 6), the PTA results included 139 bulls with evaluations computed by the ICBA in July 2019, and July 2023. The July 2019 evaluation did not include records of cows calving in 2019

^2^ ‘*a*’ is the regression intercept, ‘*b*’ is the regression coefficient, ‘r’ is the correlation, and ‘se’ stands for standard error. Asterisks indicate *p*-values of Y-intercepts and regression coefficients significantly different from zero and unity, respectively. The significance levels were ^*^, 0.01 < *P* < 0.05; ^**^, 0.001 < *P* < 0.01; and ^***^, *P* < 0.001

^3^ The averaging method was performed by taking the simple average of PTAs from the ssSNPBLUP method and from results computed by the ICBA in 2019

**Fig. 4:**
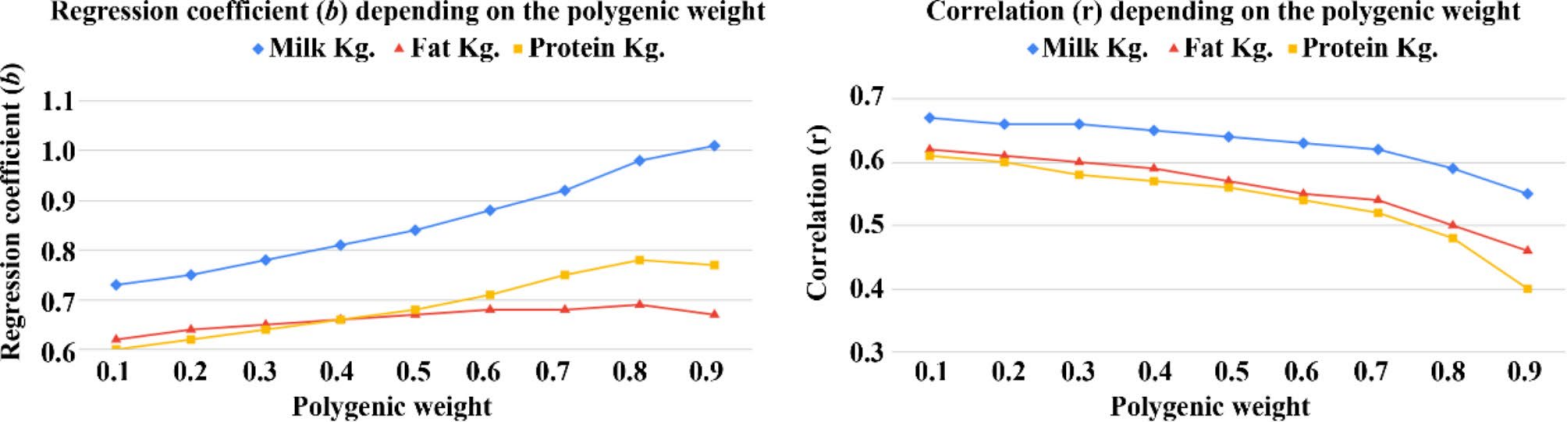
Correlation and the regression coefficients as a function of polygenic weight. PTAs were calculated from the complete dataset (DS00-23, Table 1, with daughters’ records) and regressed against the truncated dataset (DS00-19, Table 1, without daughters’ records) for the set of 141 bulls. The regression coefficients (Y-axis, left panel) and correlations (Y-axis, right panel) were plotted as a function of the assumed polygenic weight (0.1–0.9, X-axis)

Correlation and the regression coefficients as a function of polygenic weight for the ssSNPBLUP methods are given in Fig. 4. For all of the experiments, the regression coefficients tended to increase with increasing polygenic weight, whereas the corresponding correlation coefficients tended to decrease (Fig. 4, Tables S2-S3). These inverse trends between the correlation and regression coefficient values were significant (*P* < 0.05 for the three traits; -0.96, -0.76, and − 0.76 for milk, fat, and protein kg, respectively). That is, the higher the correlation is, the lower the regression coefficients and the greater the overestimation of the predicted PTAs for high evaluation bulls. For all values of polygenic weight, regressions for fat and protein were < 0.8. Similar results for the effect of changes in polygenic weights were found by Aguilar et al. [4].

### Reliabilities

Using the inverse of the mixed-model coefficient matrix, exact reliabilities for the gEBVs of the set of 141 bulls were calculated from the truncated and complete datasets for experiment 3 (Table 3). For the truncated dataset (predicted, with no daughter performances, DS00-19; Table 1), the reliabilities for all traits were similar (Fig. 2). Nonetheless, on average, protein had lower reliability (0.43) than fat and milk (0.45) (Fig. 2, Table S4), which corresponds to the lower heritability for protein (Table 2). The average estimated reliability values of the two-step method calculated for a set of 139 bulls in 2019 by the ICBA were 0.54, 0.5 and 0.57 for milk, fat and protein, respectively. However, the estimated reliabilities tended to be higher than the exact reliabilities.

### The relative contribution of genotyped cows and bulls to PTA accuracy

To evaluate the contribution of genotyped cows relative to genotyped bulls, PTAs were calculated for the set of 141 bulls from dataset DS00-23 and regressed against PTAs from DS00-19 using five different fractions of randomly selected genotyped cows or bulls (0, 0.25, 0.5, 0.75, and 1), which had phenotypic data before 2019. The analysis was performed 10 times with different randomly selected genotyped animals for each fraction (Table S5). The average estimated reliabilities for the randomly selected bulls were 0.84, 0.84, and 0.82 for milk, fat and protein, respectively, when the gEBVs were calculated from DS00-19 (Table 1). Although not linear, the increase in the number of genotyped cows or bulls improved the correlation (Table 4). Therefore, assessing the per-individual contribution to the progress in correlation values by comparing the edge values (i.e., all cows versus no cows, all bulls versus no bulls) enabled the estimation of the individual bull/cow contribution to the correlation coefficients; and dividing the average per-individual contribution of a bull by that of a cow for each trait, we calculated that each bull is equivalent to 1.8, 5, and 3.2 cows for milk, fat, and protein, respectively (Table 4). We also tested predictions using all the cow genotypes with half of the younger/older bulls. For the older bulls (*n* = 735), which had an average age of 30 years, the correlations of the predicted evaluations with the current evaluations were 0.61, 0.51 and 0.47; whereas for the younger bulls (*n* = 736), which had an average age of 16 years, the correlations were 0.62, 0.55 and 0.52 for milk, fat, and protein, respectively.

**Table 4:** Effect of different proportions of genotyped cows (a) and bulls (b) on correlations^1^

A				b			
Cows genotyped: number (ratio)	Milk	Fat	Protein	Bulls genotyped: number (ratio)	Milk	Fat	Protein
ABLUP (no genotypes)	0.4	0.35	0.18	ABLUP (no genotypes)	0.4	0.35	0.18
only bulls	0.54	0.52	0.45	only cows	0.59	0.5	0.46
976 (0.25)	0.58	0.52	0.49	276 (0.25)	0.61	0.55	0.49
2604 (0.5)	0.6	0.53	0.52	736 (0.5)	0.61	0.55	0.49
3905 (0.75)	0.62	0.55	0.54	1103 (0.75)	0.63	0.56	0.53
5207	0.64	0.57	0.56	1471	0.64	0.57	0.56

^1^ Correlations between PTAs, which were calculated from the DS00-23 dataset (Table 1), and PTAs, which were computed from the truncated dataset (DS00-19, Table 1) were examined for five different fractions of randomly selected genotyped cows or bulls (0, 0.25, 0.5, 0.75, and 1), which had phenotypic data before 2019. For each fraction there were ten rounds of random sampling and calculation. For all analyses, the polygenic weight was set to 0.5

## Discussion

To enhance the prediction of gEBVs in a small population with a limited number of progeny-tested bulls, we tested the prediction quality of adding genotyped cows using a single-step method, ssSNPBLUP. Although similar analyses were performed previously [20], the increase in the number of genotyped cows and the development of new computational methods, such as ssSNPBLUP, justified the re-examination of the approach. Moreover, ssSNPBLUP allows running a single-step model without the convergence rate hampering timely evaluation, and also allows obtaining conveniently prediction equations without back-solving [5]. By adding genotyping data of ~ 8200 cows to the current genotyped bulls, for sires with daughter records, we found that gEBVs calculated by the single-step method were very similar to those of the current two-step method based on combined bull data from the Israeli and Netherlands populations including ~ 5000 genotyped bulls (Fig. 1). The correlations of PTAs for young bulls with no daughter records with the PTAs of these bulls with progeny records were generally similar to those obtained by the currently used two-step method (Table 3).

For dairy cattle, previous results revealed that for a trait with a heritability of 0.30, 9.3 genotyped cows are equivalent to a single progeny-tested bull with a reliability of 0.8 [23]. As described previously, the Israeli Holstein population somewhat differs in terms of breeding objectives and selection indices [11]. For these dairy cattle, our study revealed that 1.8-5 genotyped cows are equivalent to one genotyped bull with an average reliability of 0.8, depending on the tested trait. These findings imply a greater value of genotyped cows for a small cattle population than was previously thought. Hence, in agreement with the 2.5 cows/bull equivalent, our results indicate that using the single-step method, records of ~ 13,000 genotyped cows may provide gEBVs with predictive value equivalent to two-step GBLUP with a total of ~ 5000 genotyped bulls. Therefore, the need to combine two populations with different breeding indexes, trait definitions and environmental characteristics may become unnecessary. This will provide technical benefits by simplifying the analysis and shortening the time between the evaluations. Moreover, with the rapidly increasing annual number of genotyped cows each year compared to genotyped bulls, the single-step method will likely improve more than the current evaluation, which is based solely on genotyped bulls. Due to the complexity of the ssGBLUP algorithm, and the fact that each animal has differing quantities of data, it is not possible to theoretically derive the predictive ratio between cows and bulls. Thus, only empirical results can be generated.

Excluding old records did not affect the overall prediction of PTAs. This may be due to several factors; the negligible number of genotyped animals in the early years, the fact that the bulls genotyped were a nonrepresentative sample and changes in genetic linkage relationships over time. When comparing “predicted” and “observed” PTAs as a function of polygenic weight, a notable trade-off between increased correlation and overestimation of PTA values was observed. This finding is in accordance with Aguilar et al. [4], who proposed that scaling different weights between genomic and pedigree-based relationship matrices can be “tuned” to reduce inflation in predicted gEBV with some loss in accuracy. Depending on the polygenic weight, excluding older genotypes can also reduce overestimation while retaining similar gEBV accuracy. Possibly, due to intensive selection in commercial populations, changes in allelic frequencies and the decay of linkage relationships over time, old genotype records are less representative of the current population [12, 20]. This was also noticed when using half of the younger/older bulls with daughter information for prediction. Younger genotypes gave better predictions, even though the mean number of informative daughters per bull was lower. Finally, the best gEBV prediction for all traits in terms of overestimation and accuracy was achieved by excluding genotypes older than 1995. Thus, the time interval of genotyped animals to be included in the analysis is an essential factor that should be carefully assessed per population and its unique characteristics. Although regressions < unity may not affect ranking bulls of the same age, it is critical when ranking young bulls without daughter records against bulls with daughter records. Interestingly, we obtained the best predictions by averaging the evaluations of the two-step and ssSNPBLUP methods. It is clear that both methods have differing limitations. Thus, averaging the two methods seemed a reasonable option. This suggests that prediction can benefit from combining methods. As both methods have differing limitations, averaging the two methods is a reasonable approach and could be possible for implementation considering that evaluations costs should be negligible compared to the costs of data generation. It should be noted that this study is based on the analysis of a single population for the three milk production traits. Thus, the replicability of the results for other populations and traits cannot be ascertained.

## Conclusions

The genotyping of approximately 1.8, 5, and 3.2 cows yielded a statistical power of one bull for milk, fat and protein, respectively. This indicates that cow genotypes are crucial to increase the size of the training set of small, genotyped populations. Excluding old genotypes improved gEBV predictions, even though the number of genotyped bulls excluded was only 5% of the total, showing that old genotypes lose their relevance for gEBV predictions. Tuning the polygenic weight and the time range of data sampling was beneficial for improving gEBV accuracy and reducing overestimation; and thus, should be part of any model calibration. However, the overestimation of bulls with high evaluations remains an issue when using a single-step method. Combining single-step and two-step methods may improve predictions and we propose to use this for future implementations.

## Materials and methods

### Animal data

Data on pedigree records, phenotypes, and genomic records were obtained for Israeli Holstein cows that calved between 2000 and 2023. Records of cows that calved after 2019 were excluded for testing future prediction accuracy. Additional extended databases included records from cows that calved between 1990 and 2023 (Table 1). Phenotype records were obtained for 305 days of milk (kg), fat (kg) and protein (kg) production as previously described [22].

### Marker data

All genotyping was performed by Neogen (Lansing, MI, USA) and using Illumina BeadChips (Illumina Inc., San Diego, CA, USA) on hair or semen samples collected in Israel by SION, the Israeli AI Institute (http://www.sion-israel.com/english/). Since genotyping of samples was performed using several SNP chip platforms with different qualities and coverage, we performed imputation using the findhap.f90 v3.0 program software [24] on all Holstein samples with genomic evaluations. A total of 50,392 genetic markers were selected for further analysis, as described in [25]. After imputation and using PLINK v1.07 [26] to filter out SNPs with minor allele frequencies (MAFs) of less than ≥ 0.05, a total of 39,669 markers per genotype were obtained.

### Linear regression analysis

Data of linear trend was fitted by least squares method using the following equation:

$$\:y=a+bx$$

where $\:y$ is an observed trait value, $\:a\:$is the intercept bias, $\:b$ is the regression coefficient, and $\:x$ is the predicted trait value.

### The ssSNPBLUP method

For all the proposed analyses, APEX- Linear Mixed Model Software was used (https://ghpc.ai). The ssSNPBLUP model [5, 6] was applied as follows:

$$\:y=X\beta\:+u+e$$

where $\:y$ is a vector of trait values for all animals and $\:\beta\:$ is a vector of all fixed effects, including herd × year × season and lactation number × farm type (kibbutz/moshav). $\:X$ is the incidence matrix linking records of individuals to the fixed effects; u is a vector of gEBV for the animals for all traits; and e is a vector of residuals.

The breeding value of trait *i* can be expressed as follows:

$$\:{u}_{i}=Zg+{a}_{i}$$

Where $\:Z$ is a design matrix containing all SNP data of the animals $\:g$ is a vector containing SNP effects and $\:{a}_{i}$ is the residual polygenic effect vector for trait *i.*

Evaluations were calculated for each trait (milk kg, fat kg, and protein kg) in a separate analysis. However, all models were “multitrait” in the sense that for every model, the parities were considered as separate traits with differing additive genetic and residual variance components. The genetic and environmental matrices among the 5 parities were estimated by a restricted maximum likelihood (REML) analysis, as described in the “Variance component analysis” section. The genetic groups of individuals with unlisted parents were determined using previously described methodology [27]. The genetic groups were based on the sex of the animal, the year of birth and the missing parent, i.e., sire, dam or both. The G matrix was adjusted to A for compatibility of the two matrices [28].

The five-lactation model was implemented as follows: The EBVs from the five-lactations of an individual were combined into a total breeding value (BVT) using the index of Weller and Ezra [22] after normalization to the mean EBV of cows born in 2010:

$$\eqalign{BVT= & {(EBV}_{1}+\:{0.73EBV}_{2}+{0.51EBV}_{3} \cr & +{0.34EBV}_{4}+{0.21EBV}_{5})/2.79}$$

### Polygenic weight

The polygenic weight is a number that summarizes the fraction of the additive genetic variance not explained by the markers. A value for this parameter must be supplied by the user of the APEX software. To determine the effect of the polygenic weight values between 0.1 and 0.9 were tested in steps of 0.1.

### Pedigree based BLUP method - ABLUP

Genetic evaluations were also computed by a standard multi-trait animal model BLUP algorithm with each parity considered a separate trait, and compared to the ssSNPBLUP method results. The model was as follows:

$$\:y=X\beta\:+Z\gamma\:+e$$

where $\:y$ is a vector of trait values for all animals and $\:\beta\:$ is a vector of all fixed effects, including herd × year × season and lactation number × farm type (kibbutz/moshav). $\:X$ is the incidence matrix linking records of individuals to the fixed effects; $\:Z$ is the incidence matrix for the breeding value vector $\:\gamma\:$; and $\:e$ is a vector of residuals.

### Variance component analysis

A sample of 390,897 records, including all records of cows born later than 2010 was selected to compute variance components for each trait. A four-generation pedigree file was constructed for the cows in this dataset, including bulls born from 2000 to 2023. REML estimates of the variance components were computed using APEX. The models were the same as the ssSNPBLUP models described above, without genomic data or genetic groups.

### Testing the models’ prediction of genetic evaluations

The models’ predictions were tested using a set of 141 sires whose daughters first calved between 2019 and 2023. A regression line was fitted between the PTAs (gEBVs/2) calculated for these bulls based on the complete data and the PTAs calculated from the corresponding truncated data (up to 2019, Table 1). We conducted seven experiments for forward prediction using different methods and datasets. This included datasets with genotypes and animal records from different time spans testing the effects of excluding old phenotypes (recorded before 1990) or old genotypes (obtained before 1995 and 2000). These experiments are fully described with the results for a polygenic weight of 0.5 in Table 3. Experiments 1–5 were solely based on the Israeli population, experiment 6 combined the Dutch and Israeli populations, and experiment 7 was an average of predictions from Experiment 6 and Experiment 3 (Table 3).

### Evaluating cow and bull contributions

Several datasets that included different fractions of genotyped cows born between 2000 and 2019 were generated to test the contribution of cows to the prediction. The datasets were produced by randomly omitting cow genotypes from the total genotype data to produce the following sets: only bulls, 0.25 of cows, 0.5 of cows, 0.75 of cows and all cows. To avoid sampling issues, for each fraction, 10 datasets were produced, each with a different random selection of individuals. Similarly, datasets with the same fractions of bull samples were also formed. An ssSNPBLUP model was run for each of these datasets and PTA predictions were tested in the same way as described in the previous paragraph using the same set of 141 sires. Thus, the additional datasets for analysis, DS00-19 and DS00-23, were generated by omitting the genotypes of individuals born before 1995 or 2000.

### Reliabilities

The exact reliabilities (*r*^2^) from the 2000–2019 dataset (Table 4) were calculated as follows:

$$\:{r}^{2}=1-\frac{w{P}_{i}w{\prime\:}}{wGw{\prime\:}\times\:diag\left({H}_{i}\right)}$$

where *P*_*i*_ is the within-individual error for the *i*^th^ individual, *diag(H*_*i*_) is the diagonal element of the H relationship matrix for the *i*^th^ individual, *w* is the lactation weight using the index described by Weller and Ezra [22], and G is the genetic variance matrix of the trait. *P* and *diag(H)* were calculated using APEX for the inversion of the mixed model coefficient matrix option for a model without genetic groups.

## Electronic supplementary material

Below is the link to the electronic supplementary material.

Supplementary Material 1

## Data Availability

The data that support the findings of this study are available from the authors, but restrictions apply to the availability of these data, which were used under license from the ICBA for the current study, and so are not publicly available. Data are, however, available from the authors upon reasonable request and with permission from the ICBA https://akol.co.il/icbaapp/mivhanparim/. For data requests, please contact AC or MG.
